# Partial nephrectomy versus radiofrequency ablation in patients with cT1a renal cell carcinoma: A surveillance, epidemiology, end results (SEER) analysis

**DOI:** 10.1097/MD.0000000000040721

**Published:** 2024-11-29

**Authors:** Bo Yang, Yang Zheng, Mengqin Zheng, Dong Wang, Shangqing Ren, Jingzhi Tian

**Affiliations:** a Department of Pediatric Surgery, Sichuan Provincial People’s Hospital, School of Medicine, University of Electronic Science and Technology of China, Chengdu, China; b School of Medicine, University of Electronic Science and Technology of China, Chengdu, China; c Robotic Minimally Invasive Surgery Center, Sichuan Provincial People’s Hospital, School of Medicine, University of Electronic Science and Technology of China, Chengdu, China; d Cancer Center, Sichuan Provincial People’s Hospital, University of Electronic Science and Technology of China, Chengdu, China.

**Keywords:** partial nephrectomy, radiofrequency ablation, renal cell carcinoma, tumor size

## Abstract

Radiofrequency ablation (RFA) has been proposed for T1a renal cell carcinoma (RCC). The present study compared partial nephrectomy (PN) with RFA for T1a RCC stratified by tumor sizes. We selected patients with RCC and underwent PN or RFA through the surveillance, epidemiology, end results (SEER) database. The Kaplan–Meier method and Cox proportional hazards regression model were conducted. Inverse probability of treatment weights was conducted for sensitivity analysis. We enrolled 15,692 patients in the unmatched cohort, 15,392 (98.1%) underwent PN, and 300 (1.9%) underwent RFA. For tumor ≦ 2 cm, PN was equal to RFA in terms of overall survival (OS) (*P* > .05) and cancer-specific survival (CSS) (*P* > .05). For tumor size 2 to 3 cm, PN is likely to have a better OS (*P* < .05)and comparable CSS (*P* > .05). For > 3 cm tumor, PN might be associated with higher OS (*P* < .05) and CSS (*P* < .05) compared with RFA. In conclusion, PN had a similar OS and CSS compared with RFA in tumor size ≦ 2 cm, RFA could be offered for elderly or patients with comorbidity. For > 2 cm tumors, RFA is not recommended. However, further randomized controlled trials are further required to validate our results.

## 1. Introduction

Renal cell carcinoma (RCC) is one of the most common urological cancers, accounting for 2% to 3% of all malignancies, with an estimated approximately 0.4 million new cases and 175,000 deaths worldwide.^[1]^ In the past several decades, because of the widespread application of various image technology, the incidental detection rates of small renal masses have been progressively increasing in patients without symptoms.^[2,3]^ The small renal masses sized <4 cm (stage T1a) accounts for 48% to 66% of all renal cancers that are diagnosed.^[4]^

Historically, radical nephrectomy was regarded as the standard treatment for RCC. With the progress in surgical techniques, nephron-sparing procedures have developed well, with a better renal function preservation.^[5]^ Partial nephrectomy (PN) was diffusely performed and has better preservation of renal function and comparable oncologic outcomes compared with radical nephrectomy, considered as the standard therapy for T1a RCC.^[6–9]^ Besides, several other nephron-sparing procedures have been proposed, for example, radiofrequency ablation (RFA).^[6,10]^ The ablation techniques are fit for those with comorbidities and elderly patients and have several advantages such as no need to clamp vessels and resect the renal parenchyma compared with PN.^[6,10,11]^ Several studies have reported comparable survival outcomes between PN and RFA, while which treatment is better in oncological outcomes remains unclear.^[12–15]^

Furthermore, Abdel-Rahman observed that the primary tumor size affects the survival for patients with small renal cancers who underwent different local treatments (surveillance, ablation, and nephrectomy).^[16]^ Therefore, according to the surveillance, epidemiology, end results (SEER) database, we compared PN with RFA in terms of oncologic outcomes for patients with T1a RCC stratified by tumor sizes.

## 2. Materials and methods

### 2.1. Data source

Data was collected via the National Cancer institute’s SEER database. The SEER registry represents accurately the U.S. population with cancers.^[17]^ The SEER database contains characteristics and data in terms of cancer incidence and survival from 18 registries. We used the data following the data use agreement with the national cancer institute.

### 2.2. Patients selection and variables

We screened patients with first primary renal cell carcinoma (RCC) diagnosed between January 1, 2004 and December 31, 2015. The patients were diagnosed with RCC by positive histology and underwent PN or RFA were included. The size of the tumor is no more than 4 cm, consistent with TNM stage T1a. We precluded the patients aged < 18 years at initial diagnosis. Patients with metastatic diseases were also excluded. As for the tumor grade, we removed the patients with the unknown tumor grade, which affect the clinical outcomes.

We used the International Classification of Diseases for Oncology, Third Edition (ICD-O-3, C64.9-Kidney, NOS) to identify renal cell carcinoma. On the basis of the American Joint Committee on Cancer 6th edition, the TNM status was categorized. As for surgery codes, the codes of PN and RFA were 30, 15, respectively.

### 2.3. Outcomes

The outcomes for this study were overall survival (OS) and cancer-specific survival (CSS). We defined the OS as the interval between initial diagnosis and all-cause deaths. We defined CSS was as the time between initial diagnosis and death associated with RCC. We estimated the OS and CSS based on the cause-of-death to site recode from SEER registry.

### 2.4. Statistical analysis

Considering the clinical characteristics, continuous and categorized variables were presented as mean with standard derivation and frequency, respectively. Student *t* test and *χ*^2^ test were carried out to evaluate the baseline differences between comparison groups. We used the Kaplan–Meier method and log-rank test to explore the difference in OS and CSS between 2 groups. Cox proportional hazards regression model adjusting for baseline characteristics were also conducted to calculate the hazard ratio (HR) and 95% confidence interval (CI). Besides, we conducted subset analyses stratified by tumor size.

Besides, we performed the sensitivity analysis to further identify the stability of the present study. We calculated the inverse probability of treatment weights (IPTW) based on the estimated propensity scores. IPTW estimates the therapy effect in a cohort whose distribution of risk factors is equivalent to that observed in all subjects.^[18]^ We carried out all statistical analyses using R (http://www.R-project.org) and Empower (R) (www.empowerstats.com, X&Ysolutions, Inc. Boston, MA). A 2-sided *P*-value < .5 was regarded as a significant difference.

## 3. Result

### 3.1. The clinical characteristics of patients

In total, 15,692 patients with T1aN0M0 as the first primary cancer were identified, of these, 15,392 (98.1%) underwent PN, and 300 (1.9%) underwent RFA. In the entire population, the patients who underwent PN were younger than the patients treated with RFA (*P* < .001), and more likely to be married (*P* = .049). Besides, the patients who underwent RFA might have a lower tumor grade (*P* < .001). The detailed information was summarized in Table 1.

**Table 1:** Clinical characteristics of the patients.

	Renal cell carcinoma (N = 15,692)		
	RFA (N = 300)	PN (N = 15392)	*P*-value
Age (years)	66.34 (12.91)	56.51 (12.32)	<.001
Tumor size (mm)	25.2 (6.89)	24.72 (8.26)	.314
Race White Black Others	236 (78.67%) 34 (11.33%) 30 (10.00%)	12,624 (82.02%) 1542 (10.02%) 1226 (7.97%)	.231
Gender Male Female	175 (58.33%) 125 (41.67%)	9175 (59.61%) 6217 (40.39%)	.656
Marital status Married Others	174 (58.00%) 126 (42.00%)	9778 (63.53%) 5614 (36.47%)	.049
Year of diagnosis 2004–2010 2011–2015	130 (43.33%) 170 (56.67%)	6408 (41.63%) 8984 (58.37%)	.554
Laterality (right)	170 (56.67%)	8023 (52.12%)	.119
Histologic subtype Clear cell Non-clear cell	199 (66.33%) 101 (33.67%)	10,221 (66.40%) 5171 (33.60%)	.979
Grade Grade I/II Grade III/IV	275 (91.67%) 25 (8.33%)	12,371 (80.37%) 3021 (19.63%)	<.001

PN = partial nephrectomy, RFA = radiofrequency ablation.

### 3.2. Comparison of RFA and PN for T1a RCC

**Figure 1.**
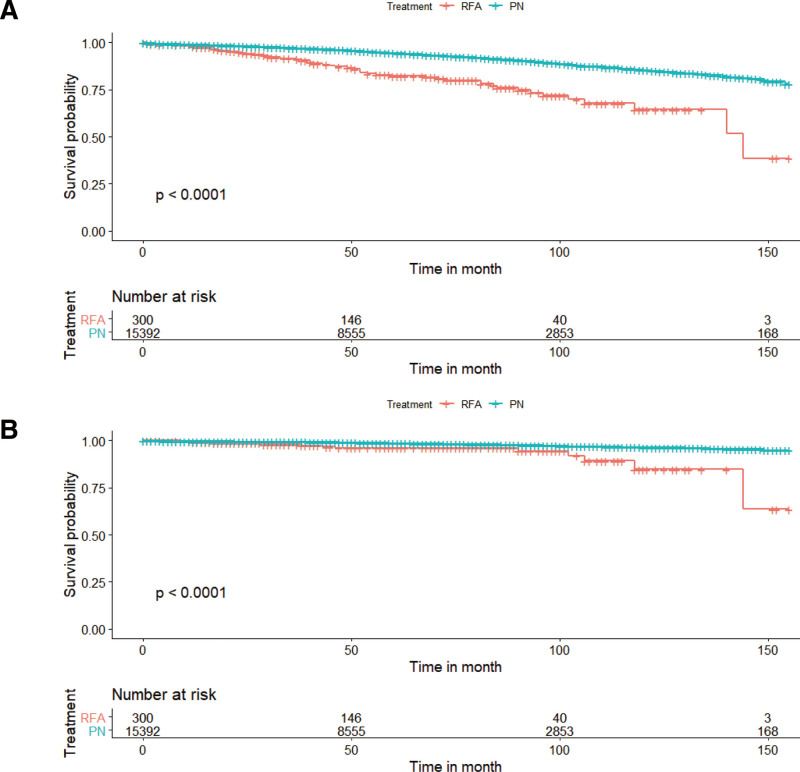
Comparison of PN and RFA for T1a RCC in terms of (A) OS; (B) CSS. CSS = cancer-specific survival, OS = overall survival, PN = partial nephrectomy, RFA = radiofrequency ablation.

**Figure 2.**
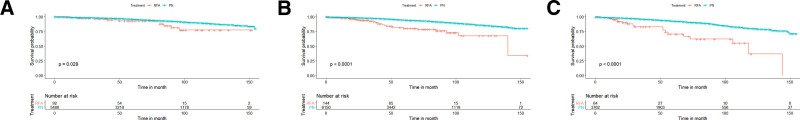
Comparison of PN and RFA in terms of OS for tumors. (A) ≦2 cm; (B): 2 to 3 m; (C) >3 cm. OS = overall survival, PN = partial nephrectomy, RFA = radiofrequency ablation.

**Figure 3.**
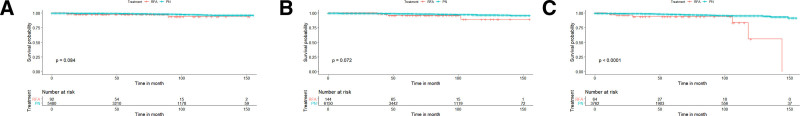
Comparison of PN and RFA in terms of CSS for tumors. (A) ≦2 cm; (B): 2 to 3 cm; (C) >3 cm. CSS = cancer-specific survival, PN = partial nephrectomy, RFA = radiofrequency ablation.

The median duration of follow-up for RFA and PN was 45.5 and 55 months in the entire cohort, respectively. The log-rank test and Cox proportional hazards regression model adjusting for race, gender, year of diagnosis, age, laterality, grade, histology, marital status, and tumor sizes showed similar results between 2 groups. In the total cohort, the patients treated with PN had a better OS (HR = 0.46; 95% CI 0.35–0.61; *P* < .001; Fig. 1A and Table 2) and CSS (HR = 0.41; 95% CI 0.23–0.72; *P* = .002; Fig. 1B and Table 2). Stratified by tumor size, for tumor size ≦ 2 cm, the patients underwent PN had a similar OS (HR = 0.68; 95% CI 0.36–1.28; *P* = .23) and CSS (HR = 0.50; 95% CI 0.16–1.61; *P* = .25) compared with the patients underwent RFA. For tumor size 2 to 3 cm, PN had a superior OS compared to RFA (HR = 0.46; 95%CI 0.30–0.70; *P* < .001), while we did not observe significant difference in CSS (HR = 0.57; 95% CI 0.21–1.57; *P* = .28) between PN and RFA. For tumor size > 3 cm, PN was better than RFA in terms of OS (HR = 0.32; 95% CI 0.20–0.52; *P* < .001; Table 2 and Fig. 2) and CSS (HR = 0.23; 95% CI 0.10–0.54; *P* < .001; Table 2 and Fig. 3).

**Table 2:** Comparison of PN and RFA in the patients with T1a RCC in Cox proportional hazards regression model.

	Multivariable		IPTW model	
	HR (95% CI)†	*P*-value	HR (95% CI)†	*P*-value
OS				
All sized T1a	0.46 (0.35–0.61)	<.001*	0.46 (0.32–0.67)	<.001*
≦2 cm	0.68 (0.36–1.28)	.23	0.93 (0.49–1.75)	.82
2–3 cm	0.46 (0.30–0.70)	<.001*	0.52 (0.32–0.84)	.008*
> 3 cm	0.32 (0.20–0.52)	<.001*	0.27 (0.15–0.50)	<.001*
CSS				
All sized T1a	0.41 (0.23–0.72)	.002*	0.59 (0.34–1.04)	.069
≦2 cm	0.50 (0.16–1.61)	.25	0.61 (0.17–2.19)	.45
2–3 cm	0.57 (0.21–1.27)	.28	0.80 (0.24–2.68)	.71
> 3 cm	0.23 (0.10–0.54)	<.001*	0.38 (0.18–0.81)	.012*

CSS = cancer-specific survival, IPTW = inverse probability of treatment weights, OS = overall survival, PN = partial nephrectomy, RFA = radiofrequency ablation.

† RFA as reference.

* *P* value < .05.

### 3.3. Sensitivity analysis

Based on the IPTW model, the patients underwent PN had an significantly increased OS (HR = 0.46; 95% CI 0.32–0.67; *P* < .001) and comparable CSS (HR = 0.59; 95% CI 0.34–1.04; *P* = .069) in Cox proportional hazards regression model adjusting for ethnicity, gender, year of diagnosis, age, laterality, grade, histology, martial status, and tumor sizes. In subset analysis, for tumor size ≦ 2 cm, RFA is not inferior to PN regarding OS (HR = 0.93; 95% CI 0.49–1.75; *P* = .82) and CSS (HR = 0.61; 95% CI 0.17–2.19; *P* = .45). Compared to RFA, PN is more likely to have a better OS (HR = 0.52; 95% CI 0.32–0.84; *P* = .008) and comparable CSS (HR = 0.80; 95% CI 0.24–2.68; *P* = .71) in the subgroup of tumor size of 2 to 3 cm. As for size > 3 cm, PN was also superior to RFA in terms of OS (HR = 0.27; 95% CI 0.15–0.50; *P* < .001) and CSS (HR = 0.38; 95% CI 0.18–0.81; *P* = .012; Table 2).

## 4. Discussion

In the present study, we compared the PN with RFA for patients with T1a RCC. For tumor size ≦ 2 cm, the RFA might be equal to PN regarding OS and CSS. For those between 2 and 3 cm, PN is more likely to have a better OS compared with RFA. For more than 3 cm tumors, PN is likely to be superior to RFA in terms of OS and CSS.

RFA is one of the newly developed therapies for small renal masses. RFA converted radiofrequency waves to heat surrounding tissue, finally resulting in coagulation necrosis and cell death.^[19]^ European Association of Urology Guidelines suggested that offer RFA to elderly or patients with comorbidities for small renal masses.^[6]^

There are several reports that compared PN with RFA in oncologic outcomes, while these results are mixed. Olweny et al included 74 patients with solitary T1a RCC and found that RFA had a comparable 5-year OS (*P* = .31) and CSS (*P* = .31).^[20]^ Liu et al observed that RFA provided comparable 10-year OS and disease-free survival to PN in clear-cell and non-clear-cell RCC in patients with T1a RCC.^[21]^ Recently, Chan et al found that T1a patients undergoing RFA had improved local recurrence-free survival (HR = 0.002, *P* = .003) and metastasis-free survival (HR = 0.002, *P* = .029) compared to PN. And RFA had a significantly smaller median decrease in eGFR postoperatively compared to PN (*P* < .001).^[22]^ Another retrospective study included 1798 patients with primary cT1N0M0 renal masses treated between 2000 and 2011 at Mayo Clinic. Among 1422 cT1a patients, 1055, 180, and 187 underwent PN, RFA, and cryoablation. RFA had a comparable local recurrence, metastasis and CSS with PN.^[23]^

Almost studies did not take the tumor sizes into consideration when evaluate the survival of patients. Using the SEER data, Tang et al observed that all-cause and cancer-specific mortality were significantly higher in >2 to 4 cm tumors than ≤2 cm tumors in patients received PN, local ablation and active surveillance.^[24]^ Generally, RFA requires safe tumor margins of 0.5 cm around RCC, thus, for a spherical tumor of 2 cm in diameter, RFA requires 9.5 cm^3^ of normal kidney tissues for ablation.^[21,25]^ And Best et al found that the outcome of RFA is strongly associated with tumor size and found that approximately 20% will recur in patients with the tumor of 3 cm or larger.^[26]^ Wah et al demonstrated that tumor size (<3 cm) is an independent factor of successful RFA in multivariate logistic regression analysis.^[27]^ Mylona et al revealed that no recurrence occurs in tumors <3 cm and technical success was 85.7%.^[28]^ Yan et al suggested that RFA might be inferior to PN for patients with the 2 to 4 cm tumor, but they did not perform the propensity analysis.^[29]^ In the present study, we found that PN was equal to RFA in tumor size of ≦2 cm. For 2 to 3 cm and >3 cm, PN had potential better survivals outcome compared with RFA. Pecoraro et al observed that relative to 2.1 to 4 cm RCCs, 0 to 2 cm RCCs harbored lower rates of high-grade tumors, lower rates of aggressive variant histology,^[30]^ which partially explain why the patients received PN and RFA had comparable survival in ≤2 cm tumor. While the managements of small renal mass still need further exploration.

As far as we know, this is the first study that compared PN with RFA for T1a RCC stratified by tumor sizes. Although we observed significant differences in several clinical characteristics between the comparison groups, we used the Cox proportional hazards regression model. Furthermore, we performed the sensitivity analysis using the IPTW model to verify the stability of our results.

However, our study is not short of defects. This study is a retrospective analysis, inherent biases might exist, such as the difference in age. Therefore, we performed sensitivity analysis. Additionally, the adjuvant therapies are unclear, which may affect the outcomes. Next, many other factors such as tumor location, approaches of surgeries are also probably associated with the prognosis. In spite of several limitations, this is the first study that compared the PN and RFA for T1a RCC stratified by tumor sizes. And our results could provide suggestions for the treatment of T1a RCC.

## 5. Conclusion

In conclusion, PN had a similar OS and CSS compared with RFA in tumor size ≦ 2 cm, RFA could be offered for elderly or patients with comorbidity. For >2 cm tumors, PN had a potential benefit in survival compared with RFA, and RFA is not recommended. However, further prospective randomized trials are required to validate our results.

## Author contributions

**Conceptualization:** Bo Yang, Mengqin Zheng, Jingzhi Tian.

**Data curation:** Bo Yang, Dong Wang, Shangqing Ren.

**Formal analysis:** Bo Yang, Yang Zheng, Mengqin Zheng, Shangqing Ren.

**Funding acquisition:** Shangqing Ren, Jingzhi Tian.

**Investigation:** Bo Yang, Yang Zheng, Mengqin Zheng, Dong Wang, Shangqing Ren, Jingzhi Tian.

**Methodology:** Bo Yang, Yang Zheng, Dong Wang.

**Project administration:** Shangqing Ren.

**Resources:** Yang Zheng.

**Software:** Bo Yang, Yang Zheng, Mengqin Zheng, Dong Wang, Shangqing Ren, Jingzhi Tian.

**Supervision:** Bo Yang, Dong Wang.

**Validation:** Yang Zheng, Dong Wang, Shangqing Ren, Jingzhi Tian.

**Visualization:** Jingzhi Tian.

**Writing – original draft:** Bo Yang, Shangqing Ren, Jingzhi Tian.

**Writing – review & editing:** Shangqing Ren, Jingzhi Tian.
